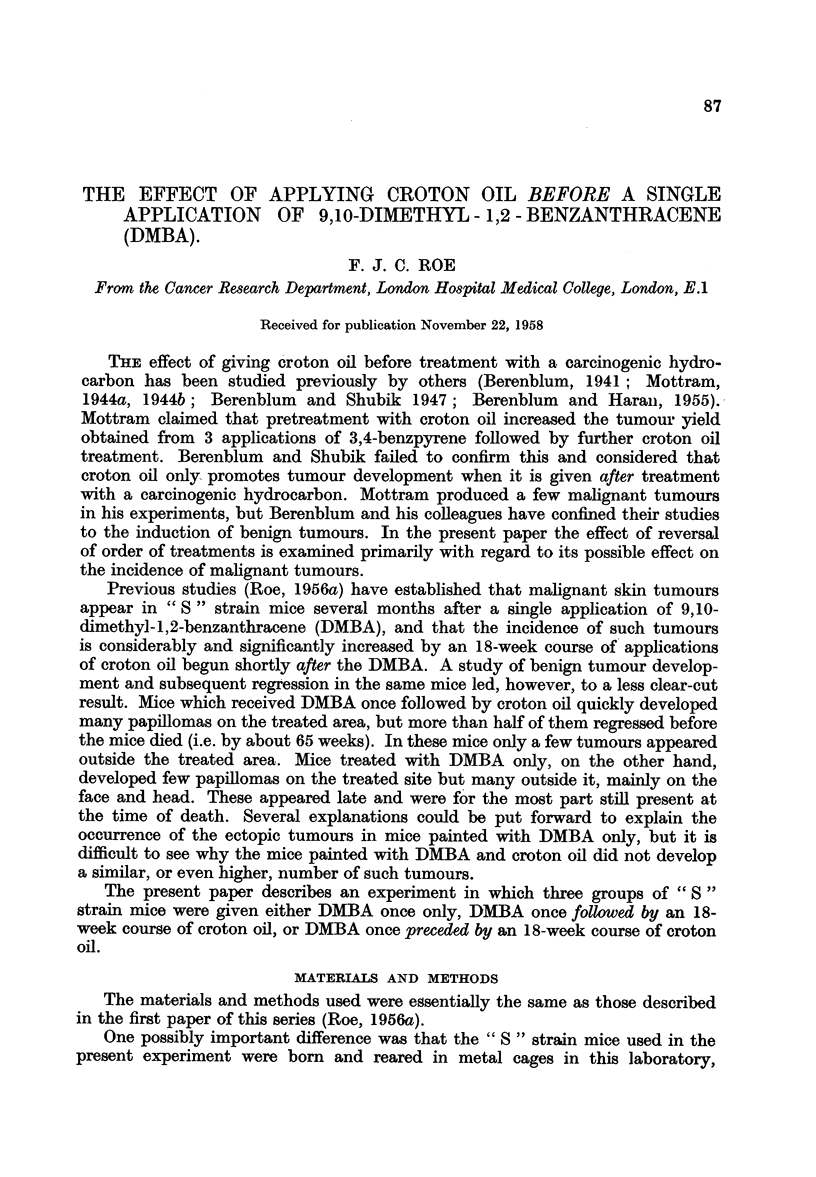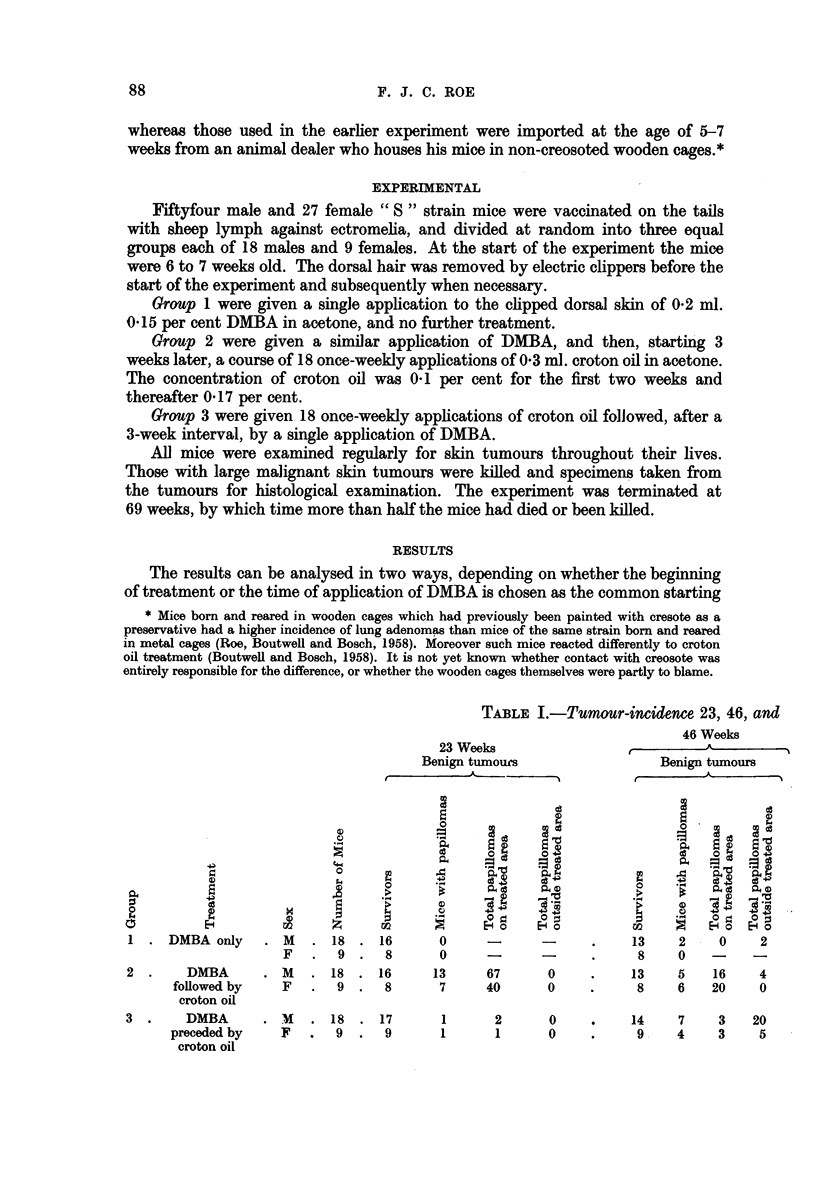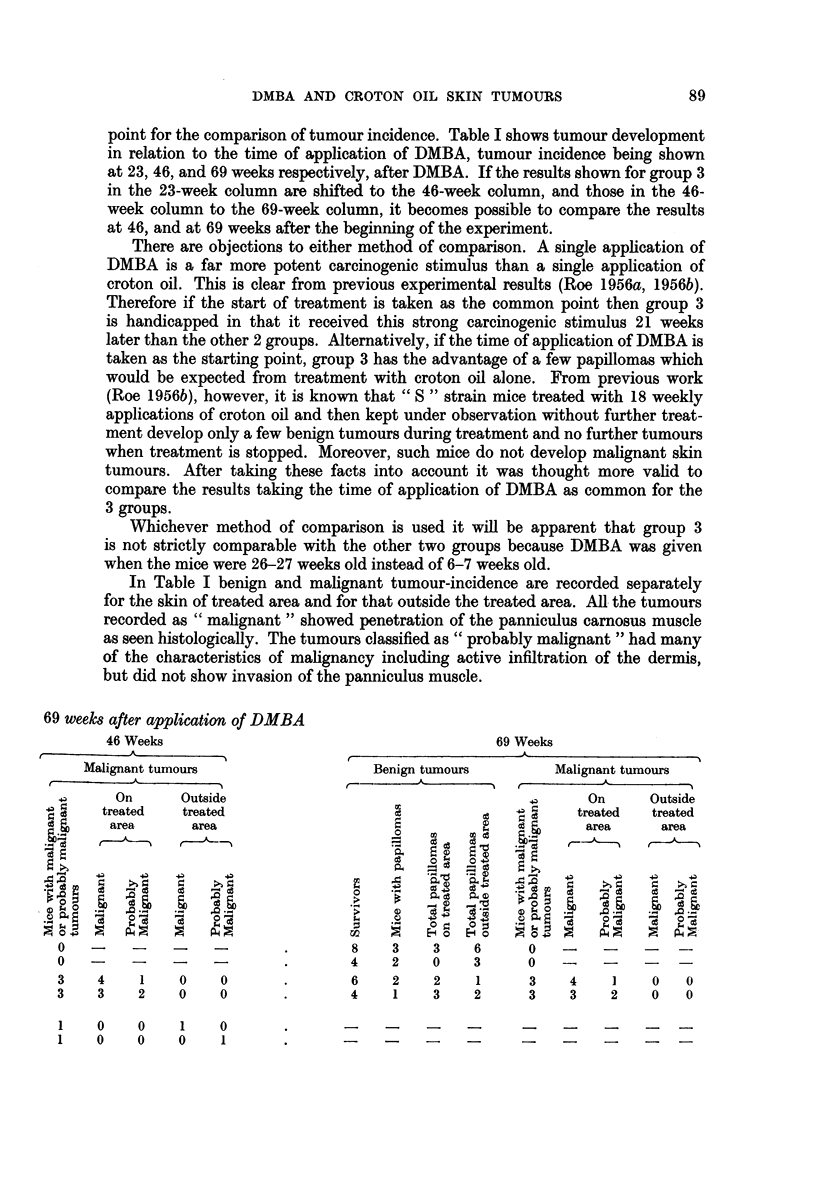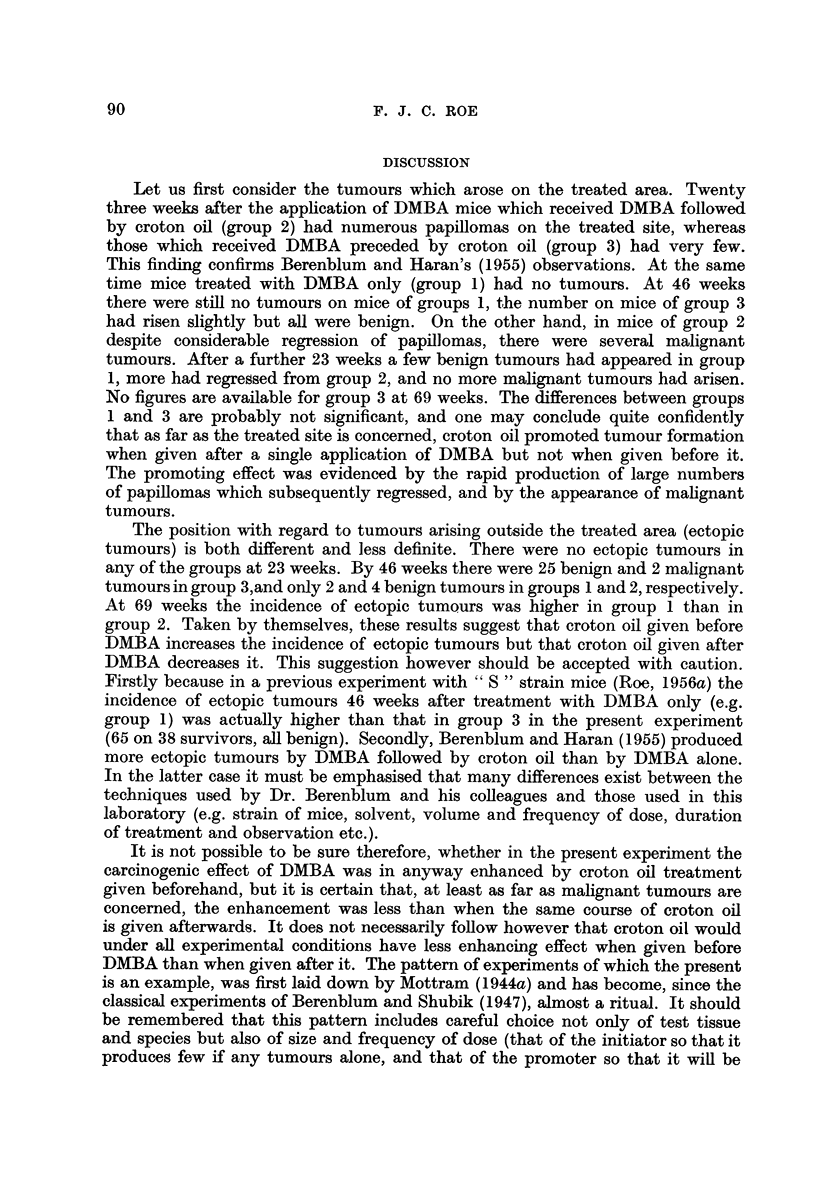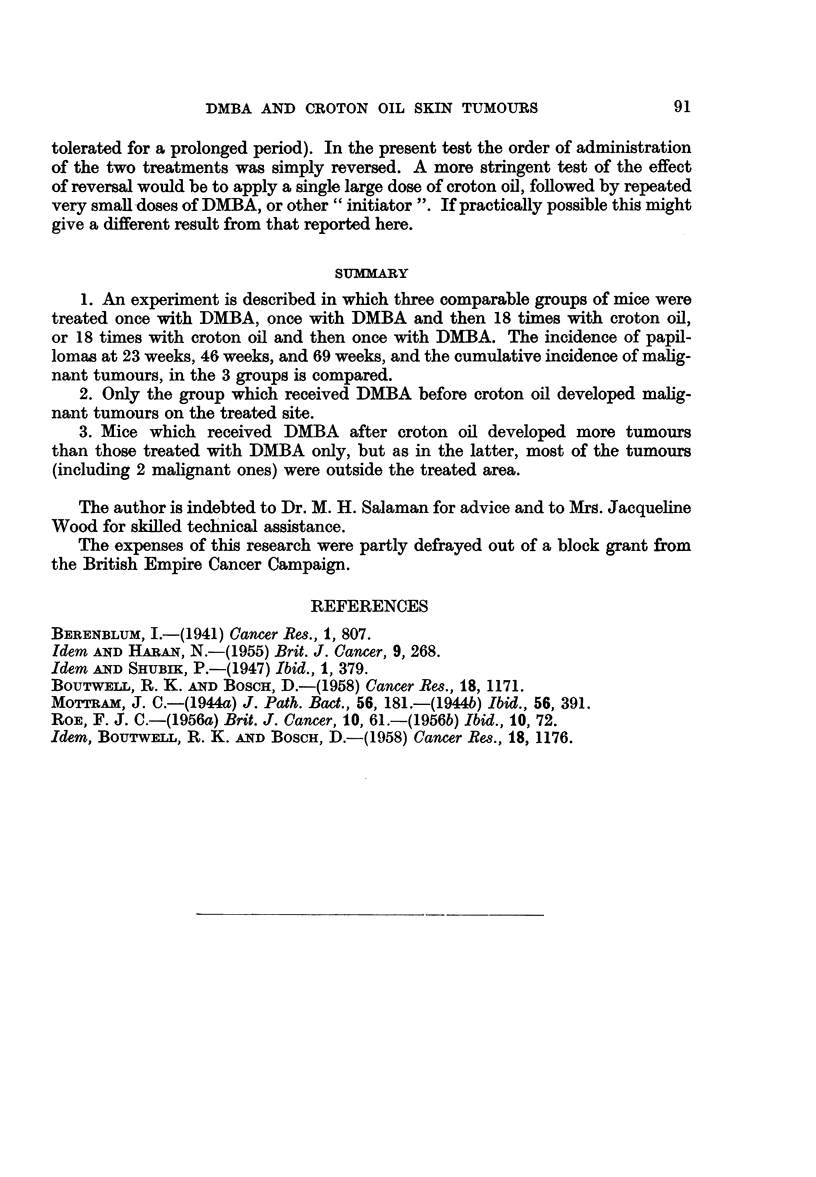# The Effect of Applying Croton Oil before a Single Application of 9,10-Dimethyl-1,2-Benzanthracene (DMBA)

**DOI:** 10.1038/bjc.1959.11

**Published:** 1959-03

**Authors:** F. J. C. Roe


### THE EFFECT OF APPLYING CROTON OIL *BEFORE* A SINGLE APPLICATION OF 9,10-DIMETHYL-1,2-BENZANTHRACENE (DMBA).

F. J. C. ROE

*From the Cancer Research Department, London Hospital Medical College, London, E.*1

Received for publication November 22, 1958

THE effect of giving croton oil before treatment with a carcinogenic hydrocarbon has been studied previously by others (Berenblum, 1941 ; Mottram, 1944*a*, 1944*b* ; Berenblum and Shubik 1947 ; Berenblum and Haran, 1955). Mottram claimed that pretreatment with croton oil increased the tumour yield obtained from 3 applications of 3,4-benzpyrene followed by further croton oil treatment. Berenblum and Shubik failed to confirm this and considered that croton oil only promotes tumour development when it is given *after* treatment with a carcinogenic hydrocarbon. Mottram produced a few malignant tumours in his experiments, but Berenblum and his colleagues have confined their studies to the induction of benign tumours. In the present paper the effect of reversal of order of treatments is examined primarily with regard to its possible effect on the incidence of malignant tumours.

Previous studies (Roe, 1956*a*) have established that malignant skin tumours appear in " S " strain mice several months after a single application of 9,10-dimethyl-1,2-benzanthracene (DMBA), and that the incidence of such tumours is considerably and significantly increased by an 18-week course of applications of croton oil begun shortly *after* the DMBA. A study of benign tumour development and subsequent regression in the same mice led, however, to a less clear-cut result. Mice which received DMBA once followed by croton oil quickly developed many papillomas on the treated area, but more than half of them regressed before the mice died (i.e. by about 65 weeks). In these mice only a few tumours appeared outside the treated area. Mice treated with DMBA only, on the other hand, developed few papillomas on the treated site but many outside it, mainly on the face and head. These appeared late and were for the most part still present at the time of death. Several explanations could be put forward to explain the occurrence of the ectopic tumours in mice painted with DMBA only, but it is difficult to see why the mice painted with DMBA and croton oil did not develop a similar, or even higher, number of such tumours.

The present paper describes an experiment in which three groups of " S " strain mice were given either DMBA once only, DMBA once *followed by* an 18-week course of croton oil, or DMBA once *preceded by* an 18-week course of croton oil.

### MATERIALS AND METHODS

The materials and methods used were essentially the same as those described in the first paper of this series (Roe, 1956*a*).

One possibly important difference was that the " S " strain mice used in the present experiment were born and reared in metal cages in this laboratory, whereas those used in the earlier experiment were imported at the age of 5–7 weeks from an animal dealer who houses his mice in non-creosoted wooden cages.*

### EXPERIMENTAL

Fiftyfour male and 27 female " S " strain mice were vaccinated on the tails with sheep lymph against ectromelia, and divided at random into three equal groups each of 18 males and 9 females. At the start of the experiment the mice were 6 to 7 weeks old. The dorsal hair was removed by electric clippers before the start of the experiment and subsequently when necessary.

*Group* 1 were given a single application to the clipped dorsal skin of 0·2 ml. 0·15 per cent DMBA in acetone, and no further treatment.

*Group* 2 were given a similar application of DMBA, and then, starting 3 weeks later, a course of 18 once-weekly applications of 0·3 ml. croton oil in acetone. The concentration of croton oil was 0·1 per cent for the first two weeks and thereafter 0·17 per cent.

*Group* 3 were given 18 once-weekly applications of croton oil followed, after a 3-week interval, by a single application of DMBA.

All mice were examined regularly for skin tumours throughout their lives. Those with large malignant skin tumours were killed and specimens taken from the tumours for histological examination. The experiment was terminated at 69 weeks, by which time more than half the mice had died or been killed.

### RESULTS

The results can be analysed in two ways, depending on whether the beginning of treatment or the time of application of DMBA is chosen as the common starting

* Mice born and reared in wooden cages which had previously been painted with cresote as a preservative had a higher incidence of lung adenomas than mice of the same strain born and reared in metal cages (Roe, Boutwell and Bosch, 1958). Moreover such mice reacted differently to croton oil treatment (Boutwell and Bosch, 1958). It is not yet known whether contact with creosote was entirely responsible for the difference, or whether the wooden cages themselves were partly to blame.

TABLE I.—*Tumour-incidence 23, 46, and*

| | | | | 23 Weeks Benign tumours | | | | 46 Weeks Benign tumours | | | |
|---|---|---|---|---|---|---|---|---|---|---|---|
| Group | Treatment | Sex | Number of Mice | Survivors | Mice with papillomas | Total papillomas on treated area | Total papillomas outside treated area | Survivors | Mice with papillomas | Total papillomas on treated area | Total papillomas outside treated area |
| 1 | DMBA only | M | 18 | 16 | 0 | — | — | 13 | 2 | 0 | 2 |
| | | F | 9 | 8 | 0 | — | — | 8 | 0 | — | — |
| 2 | DMBA followed by croton oil | M | 18 | 16 | 13 | 67 | 0 | 13 | 5 | 16 | 4 |
| | | F | 9 | 8 | 7 | 40 | 0 | 8 | 6 | 20 | 0 |
| 3 | DMBA preceded by croton oil | M | 18 | 17 | 1 | 2 | 0 | 14 | 7 | 3 | 20 |
| | | F | 9 | 9 | 1 | 1 | 0 | 9 | 4 | 3 | 5 |

point for the comparison of tumour incidence. Table I shows tumour development in relation to the time of application of DMBA, tumour incidence being shown at 23, 46, and 69 weeks respectively, after DMBA. If the results shown for group 3 in the 23-week column are shifted to the 46-week column, and those in the 46-week column to the 69-week column, it becomes possible to compare the results at 46, and at 69 weeks after the beginning of the experiment.

There are objections to either method of comparison. A single application of DMBA is a far more potent carcinogenic stimulus than a single application of croton oil. This is clear from previous experimental results (Roe 1956*a*, 1956*b*). Therefore if the start of treatment is taken as the common point then group 3 is handicapped in that it received this strong carcinogenic stimulus 21 weeks later than the other 2 groups. Alternatively, if the time of application of DMBA is taken as the starting point, group 3 has the advantage of a few papillomas which would be expected from treatment with croton oil alone. From previous work (Roe 1956*b*), however, it is known that " S " strain mice treated with 18 weekly applications of croton oil and then kept under observation without further treatment develop only a few benign tumours during treatment and no further tumours when treatment is stopped. Moreover, such mice do not develop malignant skin tumours. After taking these facts into account it was thought more valid to compare the results taking the time of application of DMBA as common for the 3 groups.

Whichever method of comparison is used it will be apparent that group 3 is not strictly comparable with the other two groups because DMBA was given when the mice were 26–27 weeks old instead of 6–7 weeks old.

In Table I benign and malignant tumour-incidence are recorded separately for the skin of treated area and for that outside the treated area. All the tumours recorded as " malignant " showed penetration of the panniculus carnosus muscle as seen histologically. The tumours classified as " probably malignant " had many of the characteristics of malignancy including active infiltration of the dermis, but did not show invasion of the panniculus muscle.

69 *weeks after application of DMBA*

| 46 Weeks: Malignant tumours | | | | | 69 Weeks: Benign tumours | | | | 69 Weeks: Malignant tumours | | | | |
|---|---|---|---|---|---|---|---|---|---|---|---|---|---|
| | On treated area | | Outside treated area | | | | | | | On treated area | | Outside treated area | |
| Mice with malignant or probably malignant tumours | Malignant | Probably Malignant | Malignant | Probably Malignant | Survivors | Mice with papillomas | Total papillomas on treated area | Total papillomas outside treated area | Mice with malignant or probably malignant tumours | Malignant | Probably Malignant | Malignant | Probably Malignant |
| 0 | — | — | — | — | 8 | 3 | 3 | 6 | 0 | — | — | — | — |
| 0 | — | — | — | — | 4 | 2 | 0 | 3 | 0 | — | — | — | — |
| 3 | 4 | 1 | 0 | 0 | 6 | 2 | 2 | 1 | 3 | 4 | 1 | 0 | 0 |
| 3 | 3 | 2 | 0 | 0 | 4 | 1 | 3 | 2 | 3 | 3 | 2 | 0 | 0 |
| 1 | 0 | 0 | 1 | 0 | — | — | — | — | — | — | — | — | — |
| 1 | 0 | 0 | 0 | 1 | — | — | — | — | — | — | — | — | — |

### DISCUSSION

Let us first consider the tumours which arose on the treated area. Twenty three weeks after the application of DMBA mice which received DMBA followed by croton oil (group 2) had numerous papillomas on the treated site, whereas those which received DMBA preceded by croton oil (group 3) had very few. This finding confirms Berenblum and Haran's (1955) observations. At the same time mice treated with DMBA only (group 1) had no tumours. At 46 weeks there were still no tumours on mice of groups 1, the number on mice of group 3 had risen slightly but all were benign. On the other hand, in mice of group 2 despite considerable regression of papillomas, there were several malignant tumours. After a further 23 weeks a few benign tumours had appeared in group 1, more had regressed from group 2, and no more malignant tumours had arisen. No figures are available for group 3 at 69 weeks. The differences between groups 1 and 3 are probably not significant, and one may conclude quite confidently that as far as the treated site is concerned, croton oil promoted tumour formation when given after a single application of DMBA but not when given before it. The promoting effect was evidenced by the rapid production of large numbers of papillomas which subsequently regressed, and by the appearance of malignant tumours.

The position with regard to tumours arising outside the treated area (ectopic tumours) is both different and less definite. There were no ectopic tumours in any of the groups at 23 weeks. By 46 weeks there were 25 benign and 2 malignant tumours in group 3,and only 2 and 4 benign tumours in groups 1 and 2, respectively. At 69 weeks the incidence of ectopic tumours was higher in group 1 than in group 2. Taken by themselves, these results suggest that croton oil given before DMBA increases the incidence of ectopic tumours but that croton oil given after DMBA decreases it. This suggestion however should be accepted with caution. Firstly because in a previous experiment with " S " strain mice (Roe, 1956*a*) the incidence of ectopic tumours 46 weeks after treatment with DMBA only (e.g. group 1) was actually higher than that in group 3 in the present experiment (65 on 38 survivors, all benign). Secondly, Berenblum and Haran (1955) produced more ectopic tumours by DMBA followed by croton oil than by DMBA alone. In the latter case it must be emphasised that many differences exist between the techniques used by Dr. Berenblum and his colleagues and those used in this laboratory (e.g. strain of mice, solvent, volume and frequency of dose, duration of treatment and observation etc.).

It is not possible to be sure therefore, whether in the present experiment the carcinogenic effect of DMBA was in anyway enhanced by croton oil treatment given beforehand, but it is certain that, at least as far as malignant tumours are concerned, the enhancement was less than when the same course of croton oil is given afterwards. It does not necessarily follow however that croton oil would under all experimental conditions have less enhancing effect when given before DMBA than when given after it. The pattern of experiments of which the present is an example, was first laid down by Mottram (1944*a*) and has become, since the classical experiments of Berenblum and Shubik (1947), almost a ritual. It should be remembered that this pattern includes careful choice not only of test tissue and species but also of size and frequency of dose (that of the initiator so that it produces few if any tumours alone, and that of the promoter so that it will be tolerated for a prolonged period). In the present test the order of administration of the two treatments was simply reversed. A more stringent test of the effect of reversal would be to apply a single large dose of croton oil, followed by repeated very small doses of DMBA, or other " initiator ". If practically possible this might give a different result from that reported here.

### SUMMARY

1. An experiment is described in which three comparable groups of mice were treated once with DMBA, once with DMBA and then 18 times with croton oil, or 18 times with croton oil and then once with DMBA. The incidence of papillomas at 23 weeks, 46 weeks, and 69 weeks, and the cumulative incidence of malignant tumours, in the 3 groups is compared.

2. Only the group which received DMBA before croton oil developed malignant tumours on the treated site.

3. Mice which received DMBA after croton oil developed more tumours than those treated with DMBA only, but as in the latter, most of the tumours (including 2 malignant ones) were outside the treated area.

The author is indebted to Dr. M. H. Salaman for advice and to Mrs. Jacqueline Wood for skilled technical assistance.

The expenses of this research were partly defrayed out of a block grant from the British Empire Cancer Campaign.

### REFERENCES

BERENBLUM, I.—(1941) *Cancer Res.*, **1**, 807.
*Idem* AND HARAN, N.—(1955) *Brit. J. Cancer*, **9**, 268.
*Idem* AND SHUBIK, P.—(1947) *Ibid.*, **1**, 379.
BOUTWELL, R. K. AND BOSCH, D.—(1958) *Cancer Res.*, **18**, 1171.
MOTTRAM, J. C.—(1944*a*) *J. Path. Bact.*, **56**, 181.—(1944*b*) *Ibid.*, **56**, 391.
ROE, F. J. C.—(1956*a*) *Brit. J. Cancer*, **10**, 61.—(1956*b*) *Ibid.*, **10**, 72.
*Idem*, BOUTWELL, R. K. AND BOSCH, D.—(1958) *Cancer Res.*, **18**, 1176.